# Crystal Phase and Morphology Control for Enhanced Luminescence in K_3_GaF_6_:Er^3+^

**DOI:** 10.3390/nano15040318

**Published:** 2025-02-19

**Authors:** Yilin Guo, Xin Pan, Yidi Zhang, Ke Su, Rong-Jun Xie, Jiayan Liao, Lefu Mei, Libing Liao

**Affiliations:** 1 Engineering Research Center of Ministry of Education for Geological Carbon Storage and Low Carbon Utilization of Resources, Beijing Key Laboratory of Materials Utilization of Nonmetallic Minerals and Solid Wastes, National Laboratory of Mineral Materials, School of Materials Science and Technology, China University of Geosciences (Beijing), Beijing 100083, China; 2009210008@email.cugb.edu.cn (Y.G.); zyd627271@outlook.com (Y.Z.); 3019220003@email.cugb.edu.cn (K.S.); clayl@cugb.edu.cn (L.L.); 2 Fujian Key Laboratory of Surface and Interface Engineering for High Performance Materials, and State Key Laboratory of Physical Chemistry of Solid Surfaces, College of Materials, Xiamen University, Xiamen 361005, China; 3 Department of Materials Science and Engineering, Southern University of Science and Technology, Shenzhen 518055, China; 4 Institute for Biomedical Materials & Devices, Faculty of Science, University of Technology Sydney, Sydney, NSW 2007, Australia; jiayan.liao@uts.edu.au

**Keywords:** upconversion luminescence, crystal phase, morphology control, K_3_GaF_6_, luminescent materials

## Abstract

Upconversion luminescent materials (UCLMs) have garnered significant attention due to their broad potential applications in fields such as display technology, biological imaging, and optical sensing. However, optimizing crystal phase and morphology remains a challenge. This study systematically investigates the effects of phase transformation and morphology control on the upconversion luminescent properties of K_3_GaF_6_:Er^3+^. By comparing different synthesis methods, we found that the hydrothermal method effectively facilitated the transformation of the Na_x_K_3-x_GaF_6_ crystal phase from cubic to monoclinic, with Na^+^/K^+^ ions playing a key role in the preparation process. Furthermore, the hydrothermal method significantly optimized the particle morphology, resulting in the formation of uniform octahedral structures. The 657 nm red emission intensity of the monoclinic phase sample doped with Er^3+^ was enhanced by 30 times compared to that of the cubic phase, clearly demonstrating the crucial role of phase transformation in luminescent performance. This study emphasizes the synergistic optimization of crystal phase and morphology through phase engineering, which substantially improves the upconversion luminescence efficiency of K_3_GaF_6_:Er^3+^, paving the way for further advancements in the design of efficient upconversion materials.

## 1. Introduction

Upconversion luminescent materials (UCLMs) have attracted significant attention for their unique ability to emit high-energy visible light upon excitation with low-energy near-infrared photons [1]. The core feature of these materials is their multiphoton absorption mechanism, which enables the conversion of low-energy photons into high-energy photons, overcoming the limitations of conventional fluorescent materials [2,3,4]. Among them, Er^3+^ is one of the most important rare earth ions in upconversion materials, primarily due to its unique 4f electron transition levels, efficient upconversion luminescence capability, tunable visible light emission, and wide applications [5,6]. Upconversion luminescent materials containing rare-earth ions, due to their unique properties, provide broad applications in fields such as biomedical imaging, laser displays, temperature sensing, and anti-counterfeiting security [7,8,9,10]. However, achieving efficient upconversion luminescence still faces challenges, with precise control over crystal phase and morphology widely considered as key factors influencing luminescent efficiency.

Fluoride-based UCLMs have long been the materials of choice due to their low phonon energy, excellent optical transparency, and chemical stability [11,12,13,14]. Among them, NaYF_4_ is the most widely studied [15,16,17,18,19]. Studies show that the phase transition from cubic (α-phase) to hexagonal (β-phase) significantly enhances upconversion efficiency, primarily due to the higher lattice symmetry and fewer defects in the hexagonal phase [20,21,22,23]. Moreover, well-defined micro/nanoparticles with specific morphologies, such as octahedral SrF_2_, also demonstrate excellent performance in optimizing light absorption and improving photon coupling efficiency [24,25,26]. These findings highlight that phase optimization and morphology control are crucial in enhancing UCLM performance [27,28,29,30,31,32,33].

As an emerging fluoride-based upconversion material, K_3_GaF_6_ has drawn attention due to its low phonon energy and flexible crystal structure [34,35]. However, compared to traditional materials like NaYF_4_ [36,37,38,39], research on crystal phase and morphology control in K_3_GaF_6_ is still in its early stages. Previous studies have primarily focused on K_3_GaF_6_ preparation via co-precipitation, which has notable limitations in achieving phase transitions and morphology optimization [40]. Currently, there is a lack of systematic studies on how the phase transition from cubic to monoclinic affects upconversion luminescence properties, and how morphology can be fine-tuned to optimize performance. Addressing these knowledge gaps is crucial for designing efficient upconversion luminescent materials.

**Figure 1:**
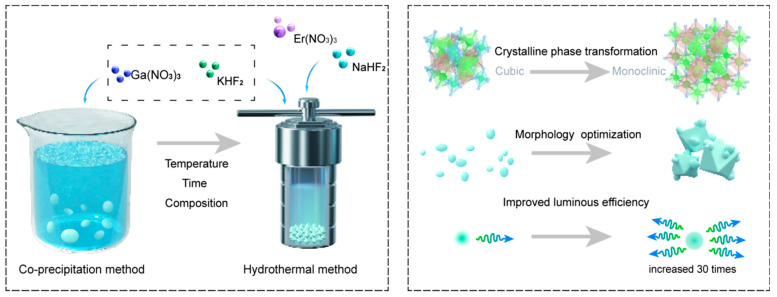
Schematic illustration of the comprehensive regulation of crystal phase and morphology through co-precipitation and hydrothermal methods. By precisely controlling reaction parameters, including the reaction temperature, time, and composition (**left**), the synergistic optimization of crystal structure and morphology was achieved, leading to significantly enhanced luminescent performance (**right**).

In this study, the synergistic effect of the crystal-phase transition and morphology optimization on the upconversion luminescence performance of K_3_GaF_6_:Er^3+^ was systematically explored. By comparing the co-precipitation and hydrothermal methods, and precisely controlling synthesis parameters, we revealed the mechanism by which the crystal-phase transition impacts luminescence performance. Furthermore, we propose a novel synthetic strategy combining crystal phase and morphology optimization (Figure 1). The monoclinic phase achieved through precise phase and morphology engineering exhibits a 30-fold enhancement in 657 nm red emission intensity compared to the cubic phase. These results underscore the critical role of structural and morphological control in improving luminescent efficiency, offering valuable insights for advancing fluoride-based UCLMs and their applications in biological imaging, optoelectronic devices, and laser displays.

## 2. Experimental Section

### 2.1. Experimental Materials

The materials used in this study include deionized water (H_2_O, laboratory), hexahydrate gallium nitrate (Ga(NO_3_)_3_·6H_2_O, Beijing Chemical Works, Beijing, China), potassium hydrogen fluoride (KHF_2_, Shanghai Aladdin Biochemical Technology Co., Ltd., Shanghai, China), erbium nitrate (Er(NO_3_)_3_·6H_2_O, Beijing Chemical Works, Beijing, China), and sodium hydrogen fluoride (NaHF_2_, Shanghai Aladdin Biochemical Technology Co., Ltd., Shanghai, China). The purchased reagents have a purity of 99.7%.

KHF_2_ and Ga(NO_3_)_3_ were used to prepare K_3_GaF_6_. NaHF_2_, KHF_2_, and Ga(NO_3_)_3_ were used to prepare Na_x_K_3-x_GaF_6_. Er(NO_3_)_3_·6H_2_O and Na_2.5_K_0.5_GaF_6_ were used to synthesize Na_2.5_K_0.5_GaF6:xEr^3+^. H_2_O was used as the solvent and for washing the samples. Details of the usage are provided in the Synthesis section.

### 2.2. Synthesis Methods

#### 2.2.1. Co-Precipitation Method for Preparing K_3_GaF_6_ Samples

The hexahydrate of gallium nitrate (Ga(NO_3_)_3_·6H_2_O) was dissolved in deionized water to prepare a solution with a concentration of 0.001 mol in 4 mL, which was set aside. Potassium hydrogen fluoride (amounts of KHF_2_ used were 0.7810 g, 2.3431 g, and 3.9052 g) was weighed according to the stoichiometric ratio and added to a PTFE-lined reaction vessel. Then, 46 mL of deionized water and a magnetic stir bar were added, and the mixture was stirred until fully dissolved. The Ga(NO_3_)_3_ solution was slowly added to the KHF_2_ solution using a micropipette, under continuous stirring to ensure uniform mixing. After stirring, the solution was allowed to stand for phase separation, and the supernatant was discarded. The precipitate was transferred to a centrifuge tube, washed three times with deionized water (the water level added each time was consistent), and centrifuged (9000 rpm, 5 min, at room temperature) after each wash. The final precipitate was dried at 60 °C for 10 h, then ground into powder, yielding the K_3_GaF_6_ sample (the amount of the powder is approximately 1 mmol) prepared via co-precipitation.

#### 2.2.2. Hydrothermal Method for Preparing K_3_GaF_6_ Samples

The required amount of KHF_2_ was weighed and placed in a polytetrafluoroethylene (PTFE) liner. Deionized water was added, and the mixture was stirred with a magnetic stirrer until fully dissolved. The pre-treated Ga(NO_3_)_3_ solution was then added gradually and stirred thoroughly. After achieving uniform mixing, the solution was transferred to a high-pressure reactor (2.5 MPa) and sealed. The reactor was placed in a constant-temperature electric blast drying oven, where the reaction temperature (e.g., 200 °C) and duration (e.g., 10 h) were set. Once the reaction was complete, the reactor was allowed to cool to room temperature. The reaction products were collected, washed with deionized water, centrifuged, and dried. The drying temperature was set at 90 °C, with a drying time of approximately 8–12 h. Finally, the sample was ground into a fine powder using a mortar, resulting in the K_3_GaF_6_ sample prepared via the hydrothermal method.

#### 2.2.3. Hydrothermal Method for Preparing Na_x_K_3-x_GaF_6_ Samples

The required amount of KHF₂ and NaHF₂ was weighed and placed in a polytetrafluoroethylene (PTFE) liner. Deionized water was added, and the mixture was stirred with a magnetic stirrer until fully dissolved. The pre-treated Ga(NO_3_)_3_ solution was then added gradually and stirred thoroughly. After achieving uniform mixing, the solution was transferred to a high-pressure reactor (2.5 MPa) and sealed. The reactor was placed in a constant-temperature electric blast drying oven, where the reaction temperature and duration were set. Once the reaction was complete, the reactor was allowed to cool to room temperature (approximately 8–12 h). The reaction products were collected, washed with deionized water, centrifuged, and dried. Finally, the sample was ground into a fine powder using a mortar, resulting in the Na_x_K_3-x_GaF_6_ sample prepared via the hydrothermal method.

#### 2.2.4. Hydrothermal Method for Preparing Na_2.5_K_0.5_GaF_6_:xEr^3+^ Samples

Due to the large errors that could arise from directly weighing the small amounts, we prepared a pre-made solution for the sample preparation. Er(NO_3_)_3_·6H₂O was dissolved in water to a concentration of 1 mmol × 0.01/2 mL, and the volume of deionized water was adjusted based on the doping amount to ensure that the total volume of the solution (50 mL) remained constant, with consistent initial concentrations of the ions.

The required amount of KHF_2_ and NaHF_2_ in a 1:5 ratio (the amount of NaHF_2_ used is 25 mmol, while the amount of KHF_2_ is 5 mmol) was weighed and placed in a polytetrafluoroethylene (PTFE) liner. Deionized water was added, and the mixture was stirred with a magnetic stirrer until fully dissolved. The pre-treated Ga(NO_3_)_3_ and Er(NO_3_)_3_·6H₂O solution was then added gradually and stirred thoroughly. The total amount of Ga(NO_3_)_3_ and Er(NO_3_)_3_ is 1 mmol, with the doping amounts of Er(NO_3_)_3_ being 0.005, 0.010, 0.015, 0.020 and 0.025 mmol. After achieving uniform mixing, the solution was transferred to a high-pressure reactor (2.5 MPa) and sealed. The reactor was placed in a constant-temperature electric blast drying oven, where the reaction temperature and duration were set. Once the reaction was complete, the reactor was allowed to cool to room temperature (approximately 8–12 h). The reaction products were collected, washed with deionized water, centrifuged, and dried. Finally, the sample was ground into a fine powder using a mortar, resulting in the Na_2.5_K_0.5_GaF_6_:xEr^3+^ (x = 0.005, 0.010, 0.015, 0.020, 0.025 mol) sample prepared via the hydrothermal method.

### 2.3. Testing Methods

The crystal structure and phase composition of the samples were analyzed using an X-ray diffractometer (D8 Advance, Bruker, Berlin, Germany). Cu Kα radiation (λ = 1.5418 Å) was used as the light source, with an operating voltage of 40 kV and a current of 40 mA. The scan range was from 2θ = 10° to 70°, with a step width of 0.02° and a scan speed of 24°/min. The measurements were performed at room temperature.

The surface morphology and particle size of the samples were observed using a scanning electron microscope (Sigma300, Zeiss, Oberkochen, Germany). The testing conditions included an emission voltage of 200 V to 30 kV, an electron beam current of 0.6 pA to 200 nA, and an energy resolution of 129.45 eV.

The optical properties of the samples were characterized using a fluorescence spectrometer (F-4700, Hitachi, Tokyo, Japan). A 980 nm external infrared light emitter was used as the excitation light source. The testing conditions were as follows: a continuous-wave laser, scanning range of 500–750 nm, a scanning speed of 240 nm/min, a current of 1 mA, a voltage of 500 V, and a power density of 1 W/mm^2^.

## 3. Results and Discussion

### 3.1. Crystal Phase and Morphology

The interplay between crystal phase and morphology plays a pivotal role in determining the functional performance of upconversion luminescent materials. Understanding how synthesis conditions influence both parameters provides valuable insights for optimizing material properties. In this section, we first focus on the phase transition and crystallinity of K_3_GaF_6_ under different preparation methods, followed by an exploration of how these methods impact particle morphology and surface characteristics. By systematically addressing these aspects, we aim to establish a comprehensive framework to enhance the structure of K_3_GaF_6_, enabling it to exhibit superior optical properties when doped with Er^3+^.

#### 3.1.1. Phase Transition and Crystallinity

To explore the effects of synthesis conditions on the phase transitions and crystallinity of K_3_GaF_6_, we systematically investigated samples prepared by co-precipitation and hydrothermal methods using X-ray diffraction (XRD).

**Figure 2:**
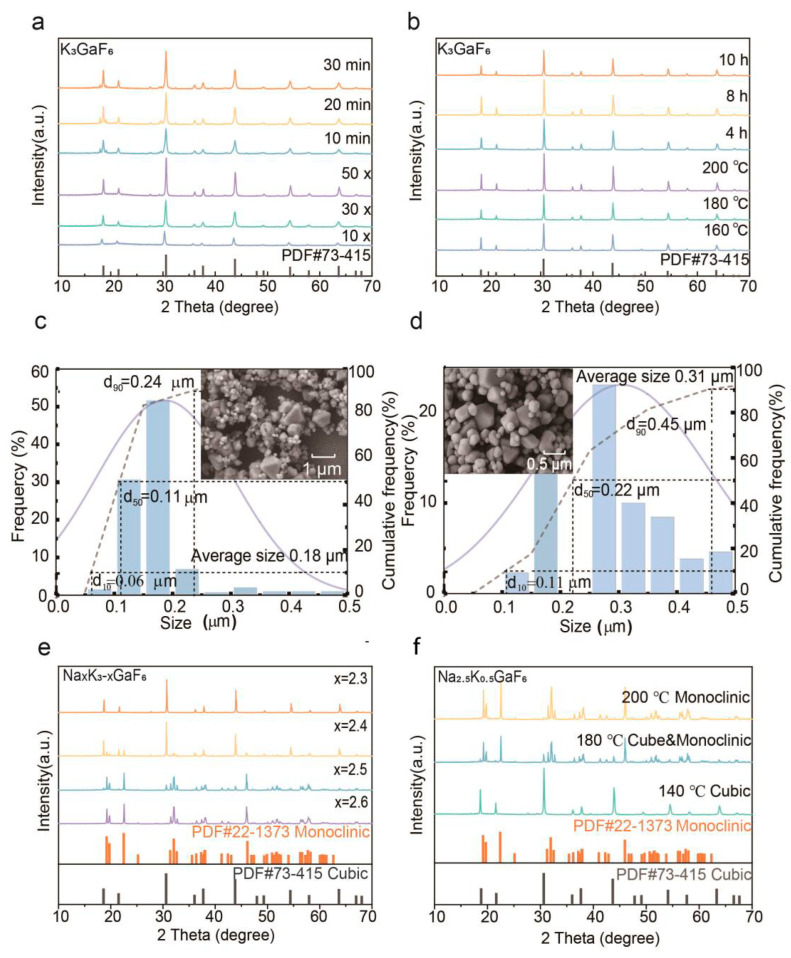
(**a**) XRD patterns of K_3_GaF_6_ samples prepared by the co-precipitation method, at stirring times of 10, 20, and 30 min, and with KHF_2_ amounts that are 10, 30, and 50 times the molar amount of Ga(NO_3_)_3_; (**b**) XRD patterns of K_3_GaF_6_ samples prepared by the hydrothermal method, with holding times of 4, 8, and 10 h, and reaction temperatures of 160, 180, and 200 °C; (**c**) the SEM images and particle size distribution of samples prepared via the hydrothermal method at 160 °C reaction temperatures; (**d**) the SEM images and particle size distribution of samples prepared via the hydrothermal method at 200 °C reaction temperatures; (**e**) XRD patterns of Na_x_K_3-x_GaF_6_ samples (x = 2.6, 2.5, 2.4, 2.3); (**f**) XRD patterns of Na_2.5_K_0.5_GaF_6_ samples prepared at different reaction temperatures (140, 180, 200 °C).

The co-precipitation method was employed to study the effects of the KHF_2_/Ga(NO_3_)_3_ molar ratio and stirring time on crystal phase formation. Samples prepared under both experimental conditions, consisting of 10, 30, and 50 times the molar ratio of KHF_2_ to Ga(NO_3_)_3_ (Table S1, Figure S1) and stirring durations of 10 to 30 min, respectively, consistently exhibited a pure cubic phase of K_3_GaF_6_ (see Figure 2a, Table S2, Figure S2). However, the XRD patterns revealed broader peaks, indicating relatively low crystallinity. This is attributed to the rapid nucleation and the limited crystal growth time during the co-precipitation process, which can result in lattice defects and internal stresses.

The hydrothermal method was utilized to overcome the limitations of co-precipitation, allowing for precise control of reaction temperature and holding time. Samples synthesized at 180 °C for 4, 8, and 10 h (Figure 2b and Figure S3, Table S3), as well as those prepared at a holding time of 10 h with reaction temperatures of 160 °C, 180 °C, and 200 °C, displayed progressively sharper XRD peaks, indicative of improved crystallinity (Table S4, Figure 2b–d). At 200 °C with a holding time of 10 h, the samples achieved the highest crystallinity and phase purity, confirming the hydrothermal method’s ability to enhance crystal growth by providing sufficient energy for atomic rearrangement.

The sample prepared by the co-precipitation method has broader diffraction peaks compared to the sample prepared by the hydrothermal method. Based on the XRD data, the crystallite sizes corresponding to the diffraction peaks at θ values of 15.15° and 21.77° were determined using the Scherrer formula (Table S5). From the results, it can be observed that the sample prepared by the co-precipitation method has smaller crystallite sizes, which is consistent with the peak broadening observed in the XRD pattern (Figure 2a). According to the Scherrer equation, smaller crystallite sizes lead to broader diffraction peaks and lower crystallinity due to the reduction in coherent scattering domains. Moreover, rapid nucleation and limited crystal growth time also contribute to lattice defects and internal stresses, which further enhance the peak broadening. For the sample prepared by the hydrothermal method, the crystallite size is larger, which typically leads to sharper peaks in the XRD pattern (Figure 2b) and higher crystallinity. This is attributed to rapid nucleation and limited crystal growth time during the co-precipitation process, which can result in lattice defects and internal stresses.

The phase transition from cubic to monoclinic was systematically studied by varying the Na⁺/K⁺ ion ratio and reaction parameters. At a reaction temperature of 180 °C, samples with Na_2.6_K_0.4_GaF_6_ to Na_2.4_K_0.6_GaF_6_ compositions exhibited a gradual transition to the monoclinic phase (Figure 2e). This highlights the critical role of the Na⁺/K⁺ ratio in stabilizing the monoclinic structure, which offers a larger unit cell volume and a more flexible coordination environment that is conducive to rare-earth dopant incorporation.

At lower temperatures (e.g., 140 °C), the cubic phase of Na_2.5_K_0.5_GaF_6_ remained thermodynamically stable, as no monoclinic phase was observed. As the temperature increased to 180 °C, a mixed phase emerged, with cubic phase peaks weakening and monoclinic peaks intensifying. Complete conversion to the monoclinic phase occurred at 200 °C, where sharper and narrower diffraction peaks indicated higher crystallinity (Figure 2f and Figure S4, Table S6). These observations suggest that the transition is driven by a combination of thermodynamic stability and crystallization kinetics, with higher temperatures providing the energy required to overcome the phase transformation barrier.

Extended holding times further revealed the kinetic effects on phase transition. For Na_2.5_K_0.5_GaF_6_ samples synthesized at 180 °C, the monoclinic phase fraction increased from 33% at 4 h to over 90% at 10 h (Figure S5). This indicates that prolonged reaction durations allow sufficient atomic rearrangements to achieve complete phase transformation.

Compared to the cubic phase, the monoclinic phase demonstrated superior structural stability and higher crystallinity. Its larger unit cell and flexible coordination environment are more favorable for incorporating rare-earth dopants, thereby enhancing luminescent properties. These findings underscore the importance of optimizing both thermodynamic and kinetic parameters to achieve desired crystal phases with enhanced performance.

#### 3.1.2. Morphology Evolution and Optimization

**Figure 3:**
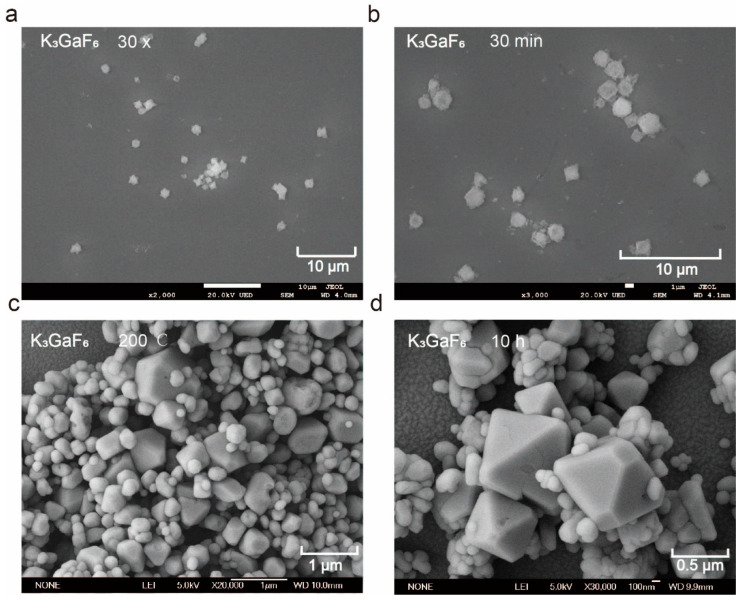
(**a**) SEM image of the K_3_GaF_6_ sample prepared by the co-precipitation method, when the amount of KHF_2_ is 30 times that of Ga(NO_3_)_3_; (**b**) SEM image of the K_3_GaF_6_ sample prepared by the co-precipitation method after stirring for 30 min; (**c**) SEM image of the K_3_GaF_6_ sample prepared by the hydrothermal method at a reaction temperature of 200 °C; (**d**) SEM image of the K_3_GaF_6_ sample prepared by the hydrothermal method after 10 h of thermal treatment.

The morphological characteristics of K_3_GaF_6_ samples were analyzed using scanning electron microscopy (SEM), revealing significant differences in grain size, shape, and surface features based on the preparation methods and reaction conditions. Samples prepared by the co-precipitation method primarily exhibited irregular spherical morphologies (Figure 3a,b). The particles, with sizes ranging from 0.50 to 3.50 µm, were loosely distributed and displayed rough, porous surfaces. This morphology likely arises from the rapid nucleation and limited crystal growth time typical of the co-precipitation process, where the quick mixing of reactants leads to simultaneous nucleation and growth. As a result, the particles were characterized by significant surface defects, which increase light scattering and potentially reduce optical performance.

In contrast, samples prepared by the hydrothermal method showed a clear progression of morphological refinement with increasing reaction temperature and holding time. At 160 °C, the particles were small spheres (~180 nm) with a relatively uniform size distribution but they retained noticeable surface defects. As the temperature increased to 200 °C, the morphology evolved into regular octahedral shapes with edge lengths of 100–400 nm and smooth surfaces, indicating a significant reduction in surface defects (Figure 3c). This transformation can be attributed to the high-temperature hydrothermal environment, which facilitates crystal surface reconstruction and promotes oriented growth along the lowest-energy crystal faces, leading to a more stable and uniform structure.

The duration of thermal treatment also played a critical role in morphological optimization. After 4 h, the particles remained irregular and agglomerated. Extending the holding time to 8 h led to the formation of partially regular octahedral morphologies with reduced but still visible defects. After 10 h, the octahedral morphology was nearly complete, with smooth surfaces and a uniform particle size distribution (Figure 3d). The average particle size increased from 225 nm to 300 nm (Figure S6), suggesting that longer reaction times allowed sufficient atomic rearrangement and stress release, resulting in enhanced crystal uniformity.

The samples prepared by the hydrothermal method demonstrated superior morphological characteristics compared to those prepared by the co-precipitation method. The irregular spherical particles obtained through co-precipitation exhibited rough surfaces and broad size distributions, limiting their optical performance due to increased light scattering. In contrast, the hydrothermal method yielded regular octahedral structures with higher crystallinity and fewer defects, thereby improving light absorption and transmission and enhancing luminescent performance. These advantages arise from the high-temperature, high-pressure conditions of the hydrothermal process, which promote energy minimization during grain growth and effectively reduce surface defects and lattice distortions.

Overall, the hydrothermal method provides significant benefits for controlling the morphology of K_3_GaF_6_, offering a pathway to optimize its structural and optical properties for advanced applications.

### 3.2. Luminescent Properties and Optimization

Building on the understanding of how crystal phase and morphology influence material properties, this section examines the luminescent performance of K_3_GaF_6_:xEr^3+^ (x = 0.005, 0.010, 0.015, 0.020, 0.025) under varying synthesis conditions and doping concentrations. By comparing the spectral characteristics of cubic and monoclinic phases and evaluating the role of Er^3+^ doping, we elucidate the mechanisms driving differences in upconversion luminescence. These findings highlight the interplay between structural optimization and luminescent efficiency, providing insights for tailoring material properties to meet specific application requirements.

#### 3.2.1. Spectral Characteristics of Cubic and Monoclinic Phases

**Figure 4:**
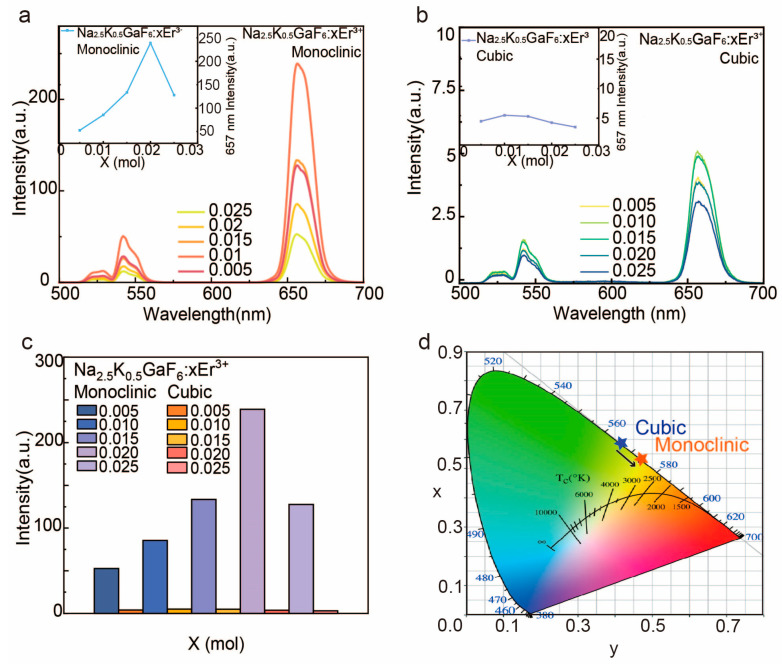
(**a**) Upconversion luminescence spectra of Na_2.5_K_0.5_GaF_6_ samples with monoclinic phases at doping concentrations of 0.5%, 1%, 1.5%, 2%, and 2.5% under the excitation of a (**a**) 980 nm laser; (**b**) upconversion luminescence spectra of Na_2.5_K_0.5_GaF_6_ samples with cubic phases at doping concentrations of 0.5%, 1%, 1.5%, 2%, and 2.5% under the excitation of a (**a**) 980 nm laser; (**c**) the bar chart showing the intensity comparison between the monoclinic phase and cubic phase at 657 nm; (**d**) CIE color coordinates for different crystal phases.

The upconversion luminescence properties of Na_2.5_K_0.5_GaF_6_ samples doped with Er^3^⁺ at concentrations of 0.5–2.5% were investigated under 980 nm laser excitation. The emission spectra (Figure 4a,b) reveal typical Er^3^⁺ transitions, including green emissions at 529 nm (^2^H_11_/_2_ → ^4^I_15_/_2_) and 542 nm ^4^S_3_/_2_ → ^4^I_15_/_2_), as well as a red emission at 657 nm (^4^F_9_/_2_ → ^4^I_15_/_2_). Notably, the monoclinic phase exhibits a red emission intensity that is approximately 30 times higher than that of the cubic phase (Figure 4c).

The lattice constant of the cubic phase is a = b = c = 10.54807 Å, representing a highly symmetrical three-dimensional structure, whereas the lattice constants of the monoclinic phase are a = 5.47336 Å, b = 5.68371 Å, and c = 7.89047 Å, with the β angle deviating from 90° (90.31231°), indicating lattice distortion. This change suggests that under controlled-temperature or chemical environments, the Na_2.5_K_0.5_GaF_6_ structure transitions from a high-symmetry cubic phase to a low-symmetry monoclinic phase. The monoclinic phase has a smaller unit cell volume (245.4609 Å^3^), indicating a more compact structure and enhanced lattice stability (Figures S7 and S8, Table S7).

In addition, in the cubic phase, Er^3+^ predominantly occupies the 4a lattice site (x = y = z = 0.000000), which is a highly symmetrical position that may lead to weaker crystal field splitting, thereby affecting the upconversion luminescence efficiency. In the monoclinic phase, Er^3+^ occupies the 2a site (x = y = z = 0.000000), but due to lattice distortion, the local coordination environment changes, enhancing crystal field splitting and increasing the transition probability of Er^3+^, thereby improving the luminescence performance (Tables S8 and S9).

The cubic phase belongs to a high-symmetry trigonal crystal system, with strong center symmetry. Er^3+^ is in a high-symmetry environment, resulting in smaller energy level splitting and reduced transition probability. In contrast, the monoclinic phase has lower symmetry, and the local crystal field distortion around Er^3+^ increases, which can enhance the electric dipole transition and improve luminescence efficiency.

Rietveld refinement data show that the unit cell volume of the cubic phase (1173.598 Å^3^) is slightly smaller than the theoretical value, indicating possible compression effects within the lattice. This internal stress may reduce the optical performance of the material. In comparison, the unit cell volume of the monoclinic phase (245.4609 Å^3^) is closer to the theoretical value, with less lattice stress, contributing to a more stable local coordination environment and improving Er^3+^ luminescence efficiency (Table S7).

The Rietveld refinement shows that the Rwp (weighted residual factor) of the cubic and monoclinic phases is 9.01751 and 0.70639, respectively. The monoclinic phase has a better fit, suggesting a more pure and stable structure, with higher refinement quality. The cubic phase may exhibit some degree of disorder, while the monoclinic phase’s structure is more stable, and this purity difference may influence the luminescence performance of Er^3+^ (Table S10).

In the cubic phase, K and Na occupy high-symmetry positions, with a uniform distribution of F, whereas in the monoclinic phase, the distribution of K and Na is uneven, indicating possible lattice defects and strain. These defects may enhance the local electric field and promote the Er^3^⁺ transition process, increasing luminescence intensity.

In the cubic phase, Er^3+^ mainly occupies the 4a site, sharing the position with Ga^3+^, with a low doping level (s.o.f. = 0.032999). This means that Er^3+^ is distributed in a high-symmetry environment with smaller crystal field splitting, which may result in lower luminescence intensity. In the monoclinic phase, Er^3+^ predominantly occupies the 2a site (s.o.f. = 0.028224). Due to the lattice distortion in the monoclinic structure, the local coordination environment around Er^3+^ becomes more irregular, potentially leading to stronger luminescence characteristics (Tables S8 and S9).

#### 3.2.2. Doping Concentration and Luminescent Efficiency

Doping concentration significantly affects the red emission intensity at 657 nm, the luminescence of cubic and monoclinic peaks at 1% and 2% doping, respectively, beyond which concentration quenching reduces efficiency. This behavior is consistent with the theory of critical dopant spacing, where excessive dopants promote cross-relaxation and non-radiative energy losses.

Quantitatively, the monoclinic phase consistently outperforms the cubic phase at all doping levels (Figure 4c). This result underscores the importance of crystal-phase optimization, as the monoclinic phase not only enhances light absorption but also reduces non-radiative losses, further improving luminescent efficiency.

#### 3.2.3. Synergistic Effects of Phase and Morphology Optimization

The superior luminescence of the monoclinic phase arises from the combined effects of phase transition and morphological refinement. The regular octahedral morphology achieved through hydrothermal synthesis reduces light scattering and enhances optical pathways, complementing the intrinsic advantages of the monoclinic crystal structure.

Luminescence tests show that the red emission intensity at 657 nm increases significantly in monoclinic samples prepared with Na^+^ and K^+^, demonstrating the impact of optimized synthesis conditions. The CIE chromaticity diagram (Figure 4d) confirms that the emission color of the monoclinic phase aligns closely with the red target gamut, indicating its suitability for applications in optoelectronic devices and biomedical imaging.

## 4. Practical Implications and Limitations

This study highlights the importance of optimizing the crystal phase and morphology of K_3_GaF_6_ in improving its upconversion luminescence properties. By systematically exploring phase transitions, doping concentrations, and morphological control, we observed that the monoclinic phase offers significant advantages over the cubic phase, particularly in red emission intensity. These improvements can be attributed to reduced lattice symmetry, enhanced energy transfer pathways, and a larger unit cell volume that facilitates rare-earth dopant incorporation. Additionally, the regular octahedral morphology achieved through hydrothermal synthesis reduces light scattering and further improves luminescent efficiency.

These findings provide valuable insights into the relationship between structural optimization and optical performance, offering potential guidance for the development of upconversion luminescent materials in applications such as biological imaging and optoelectronics. For example, the enhanced red emission intensity can improve contrast and signal-to-noise ratios in imaging applications, while the high luminescent efficiency and stability suggest potential for use in LEDs, lasers, and optical communication systems.

However, certain limitations must be acknowledged. The hydrothermal synthesis process, while effective, requires precise control of reaction conditions, which may limit scalability. Additionally, the long-term environmental stability of the monoclinic phase has yet to be thoroughly evaluated to ensure its reliability in practical applications.

Future efforts could focus on refining synthesis techniques to enhance reproducibility and scalability, as well as investigating strategies to further improve the stability and performance of K_3_GaF_6_ under varied environmental conditions. Although additional work is required, this study provides a solid foundation for understanding and optimizing the properties of K_3_GaF_6_, contributing to its potential use in advanced technologies.

## 5. Conclusions

This study elucidates the critical role of crystal-phase engineering and morphology control in the upconversion luminescence properties of K_3_GaF_6_. It demonstrates that lattice rearrangement and stress release at high temperatures are essential for forming the monoclinic phase. Optimization of the cell structure effectively reduces non-radiative transition losses. Maintaining a reaction temperature of 180 °C for 10 h concentrates the phase transition within the Na_2_._6_K_0.4_GaF_6_-to-Na_2_._4_K_0.6_GaF_6_ range, significantly enhancing crystallinity and morphological uniformity. The monoclinic-phase Er^3+^-doped sample synthesized via hydrothermal methods at 200 °C for 10 h exhibited a 30-fold increase in 657 nm red emission intensity compared to that of the cubic phase, underscoring the importance of phase optimization in improving luminescence. Furthermore, the regular octahedral morphology reduces light scattering, enhancing luminescence efficiency. This work highlights the influence of the K⁺/Na⁺ ratio on phase stability, identifies key phase transition conditions, and provides experimental insights for optimizing upconversion materials, demonstrating their potential in optical sensing and biological imaging.

## Data Availability

The data that support the plots within this paper and other findings of this study are available from the corresponding author upon reasonable request.

## Supplementary Materials

The following supporting information can be downloaded at: https://www.mdpi.com/article/10.3390/nano15040318/s1.
