# Supplementary material for: Crystal Phase and Morphology Control for Enhanced Luminescence in K3GaF6:Er3+
Source: Nanomaterials (Basel). 2025 Feb 19;15(4):318. doi: 10.3390/nano15040318 (PMC11858277; doi:10.3390/nano15040318)
Supplement: Supplementary file 1 [file nanomaterials-15-00318-s001.zip › nanomaterials-3455772-supplementary.pdf]

Supporting Information

Crystal Phase and Morphology Control for Enhanced Luminescence in $K_3GaF_6:Er^{3+}$

Yilin Guo [a,1] & Xin Pan [b,c,1], Yidi Zhang [a], Ke Su [a], Rong-Jun Xie [b,*], Jiayan Liao [d], Lefu Mei [a,*], Libin Liao [a]*

1. Beijing Key Laboratory of Materials Utilization of Nonmetallic Minerals and Solid Wastes; National Laboratory of Mineral Materials, School of Materials Sciences and Technology, China University of Geosciences, Beijing 100083, China.

2. College of Materials, Fujian Key Laboratory of Surface and Interface Engineering for High Performance Materials, and State Key Laboratory of Physical Chemistry of Solid Surfaces, Xiamen 361005, China.

3. Department of Materials Science and Engineering, Southern University of Science and Technology, Shenzhen 518055, China;

4. College of Materials, Fujian Key Laboratory of Surface and Interface Engineering for High Performance Materials, and State Key Laboratory of Physical Chemistry of Solid Surfaces, Xiamen 361005, China.

***Correspondence:**

Xin Pan (panx@sustech.edu.cn), Department of Materials Science and Engineering, Southern University of Science and Technology, Shenzhen 518055, China

Rong-Jun Xie (rjxie@xmu.edu.cn), College of Materials, Fujian Key Laboratory of Surface and Interface Engineering for High Performance Materials, and State Key Laboratory of Physical Chemistry of Solid Surfaces, Xiamen 361005, China.

Lefu Mei (mlf@cugb.edu.cn), Engineering Research Center of Ministry of Education for Geological Carbon Storage and Low Carbon Utilization of Resources, Beijing Key Laboratory of Materials Utilization of Nonmetallic Minerals and Solid Wastes, National Laboratory of Mineral Materials, School of Materials Science and Technology China University of Geosciences (Beijing), Beijing 100083, China.

This file includes Figure S1-S7, Table S1-S7

Table S1. Experimental parameters for the preparation of K_3GaF_6 samples by co-precipitation under different raw material ratio conditions

Sample ID	$Ga(NO_3)_3$ Amount /mmol	KHF_2 Amount /mmol	Stirring Time /min
1	1	10	30
2	1	30	30
3	1	50	30

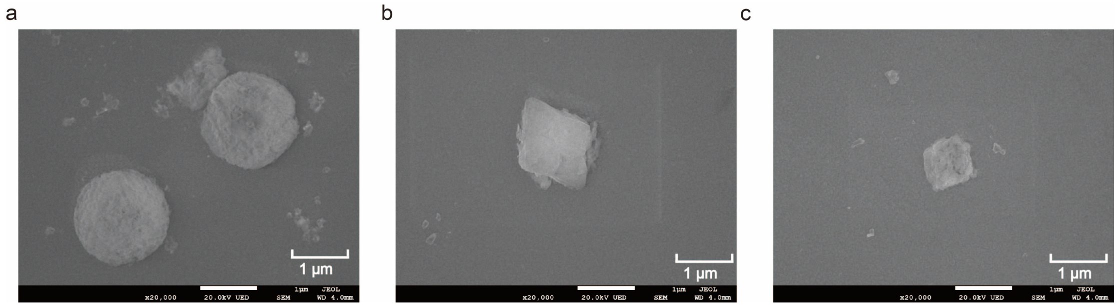

Figure S1. SEM images of samples prepared by coprecipitation with different raw material ratios: a) 10×; b) 30×; c) 50×.

Table S2. Experimental Parameters for the Preparation of K_3GaF_6 Samples via Co-precipitation Method with Different Stirring Times

Sample ID	$Ga(NO_3)_3$ Amount /mmol	KHF_2 Amount /mmol	Stirring Time /min
1	1	30	10
2	1	30	20
3	1	30	30

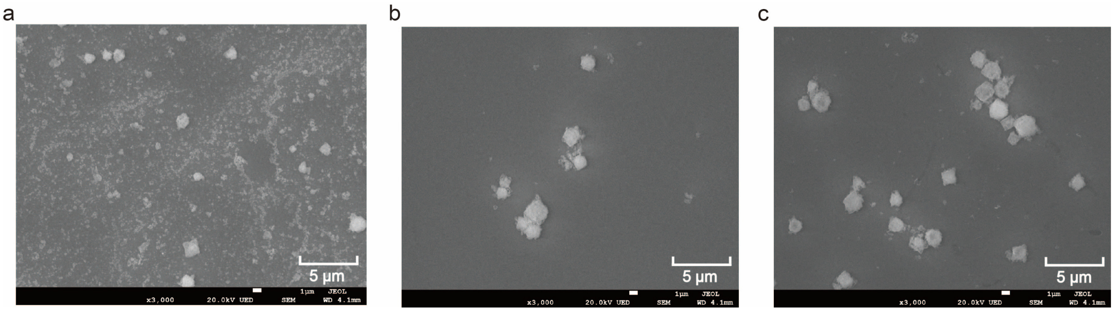

Figure S2. SEM images of samples prepared by co-precipitation method with different stirring times: a) 10 min; b) 20 min; c) 30 min.

Table S3. Experimental parameters for the synthesis of K_3GaF_6 samples by hydrothermal method with different holding times.

Sample ID	Solvent Type	Holding Time /h	Reaction Temperature /°C
1	Deionized water	4	180
2	Deionized water	8	180
3	Deionized water	10	180

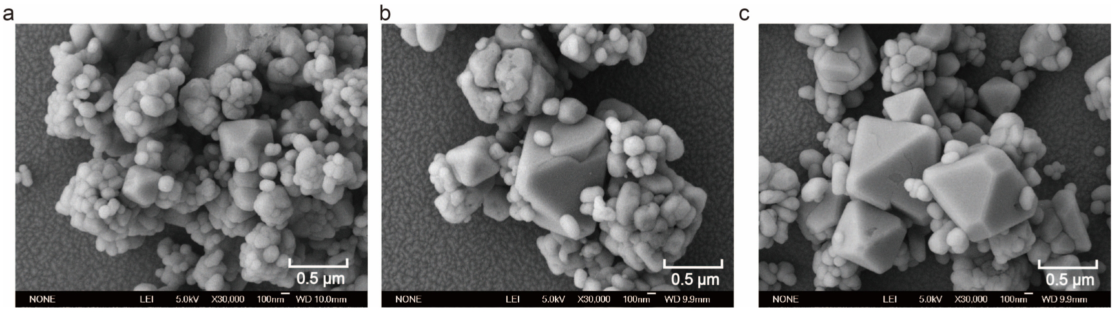

Figure S3. SEM images of samples prepared by hydrothermal method under different holding times: a) 4 h; b) 8 h; c) 10 h.

Table S4. Experimental parameters for the preparation of K_3GaF_6 samples by hydrothermal method at different reaction temperatures.

**Sample ID**	**Solvent Type**	**Holding Time /h**	**Reaction Temperature /°C**
1	Deionized water	10	160
2	Deionized water	10	180
3	Deionized water	10	200

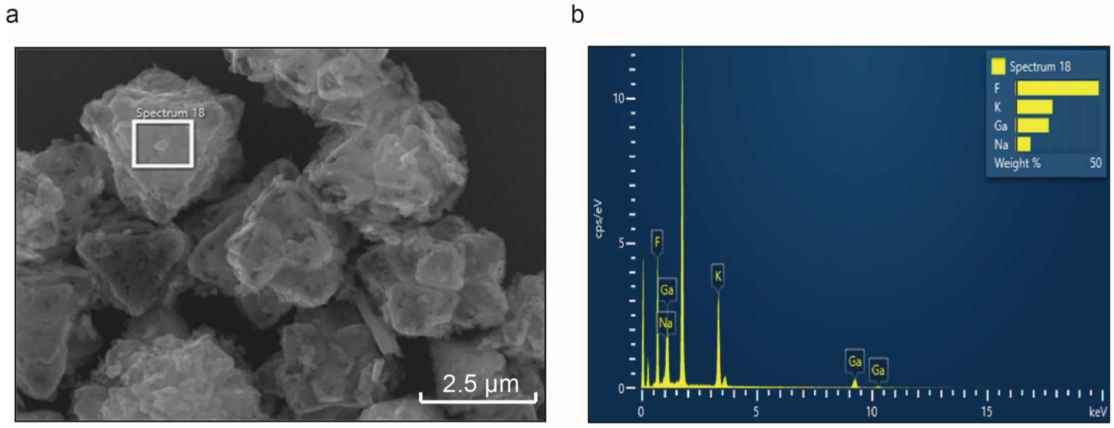

Figure S4. The EDX image of $Na_{2.50}K_{0.50}GaF_6$

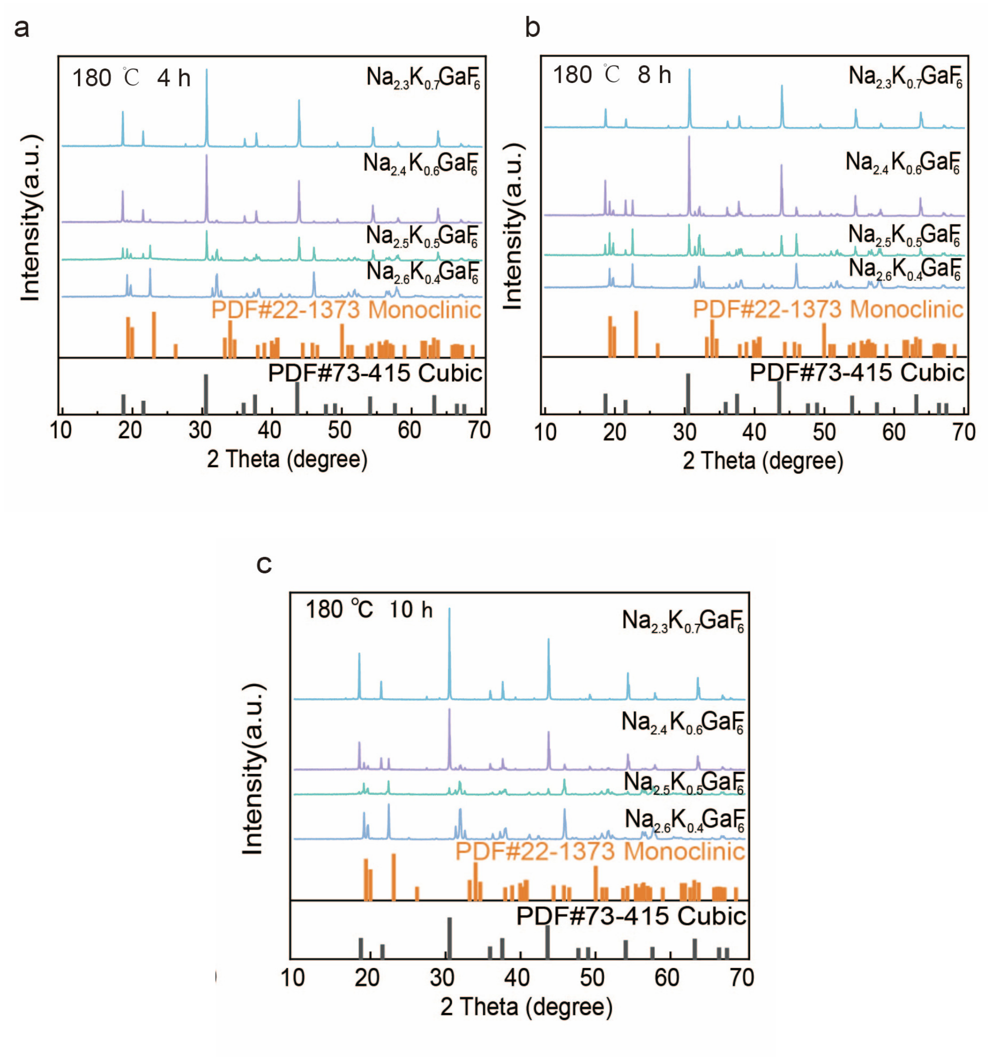

Figure S5. XRD patterns of $Na_xK_{1-x}GaF_6$ (x=2.3, 2.4, 2.5, 2.6) held at 180°C for a)4; b)8; c)10 hours.

Table S5. Comparison of crystallite sizes between the co-precipitation and hydrothermal methods.

	**The co-precipitation method**		**The hydrothermal method**	
**θ**	Preparation Conditions	Grain Size(nm)	Preparation Conditions	Grain Size(nm)
**15.15**	10x	277.98	160°C	445.29

	30x	249.21	180°C	468.81
	50x	335.81	200°C	479.9
	10min	212.2	4h	420.26
	20min	283.98	8h	448.1
	30min	280.73	10h	468.81
**21.77**	10x	244.85	160°C	344.16
	30x	199.52	180°C	369.11
	50x	278.05	200°C	387.51
	10min	168.41	4h	334.03
	20min	223.67	8h	347.39
	30min	215.22	10h	369.11

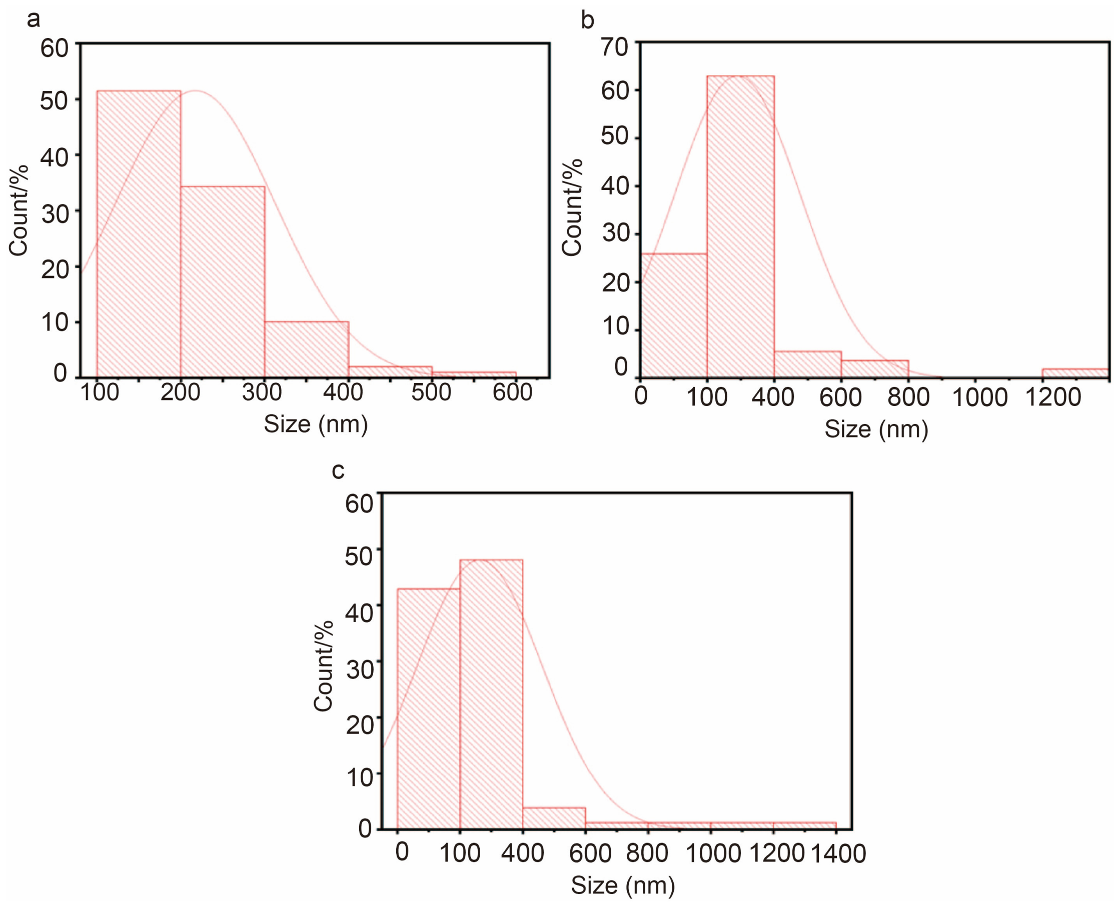

Figure S6. The particle size distributions of the samples prepared by the hydrothermal method after 4h, 8h, and 10h of heating.

Table S6. XRD patterns of $Na_{2.50}K_{0.50}GaF_6$ at 140°C, 180°C, and 200°C.

θ	Temperature (°C)	Crystallite Size (nm)	Crystal Phase
11	140	538.85	Cubic
	180	762.14	Cubic-Monoclinic Transition
	200	749.36	Monoclinic

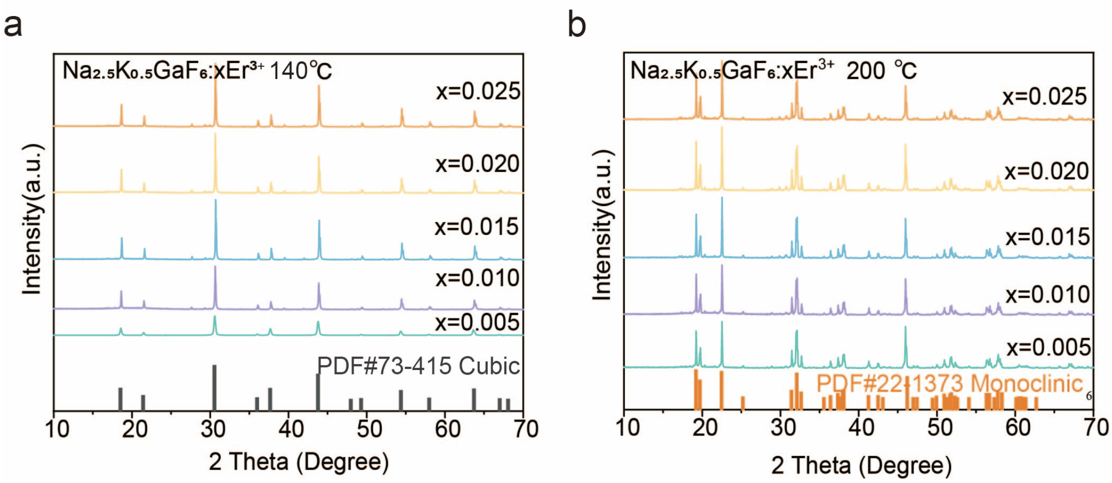

Figure S7. $Na_{2.50}K_{0.50}GaF_6$:xEr^{3+}(x=0.005, 0.010, 0.015, 0.020, 0.025) monoclinic and cubic phases XRD.

Table S7. $Na_{2.50}K_{0.50}GaF_6$:xEr^{3+} comparison of cell parameters between monoclinic and cubic phases.

Cell parameters	Cubic phase	Monoclinic phase
a	10.54807	5.47336
b	10.54807	5.68371
c	10.54807	7.89047
alpha	90	90
beta	90	90.31231
gamma	90	90
Volume	1173.598	245.4609

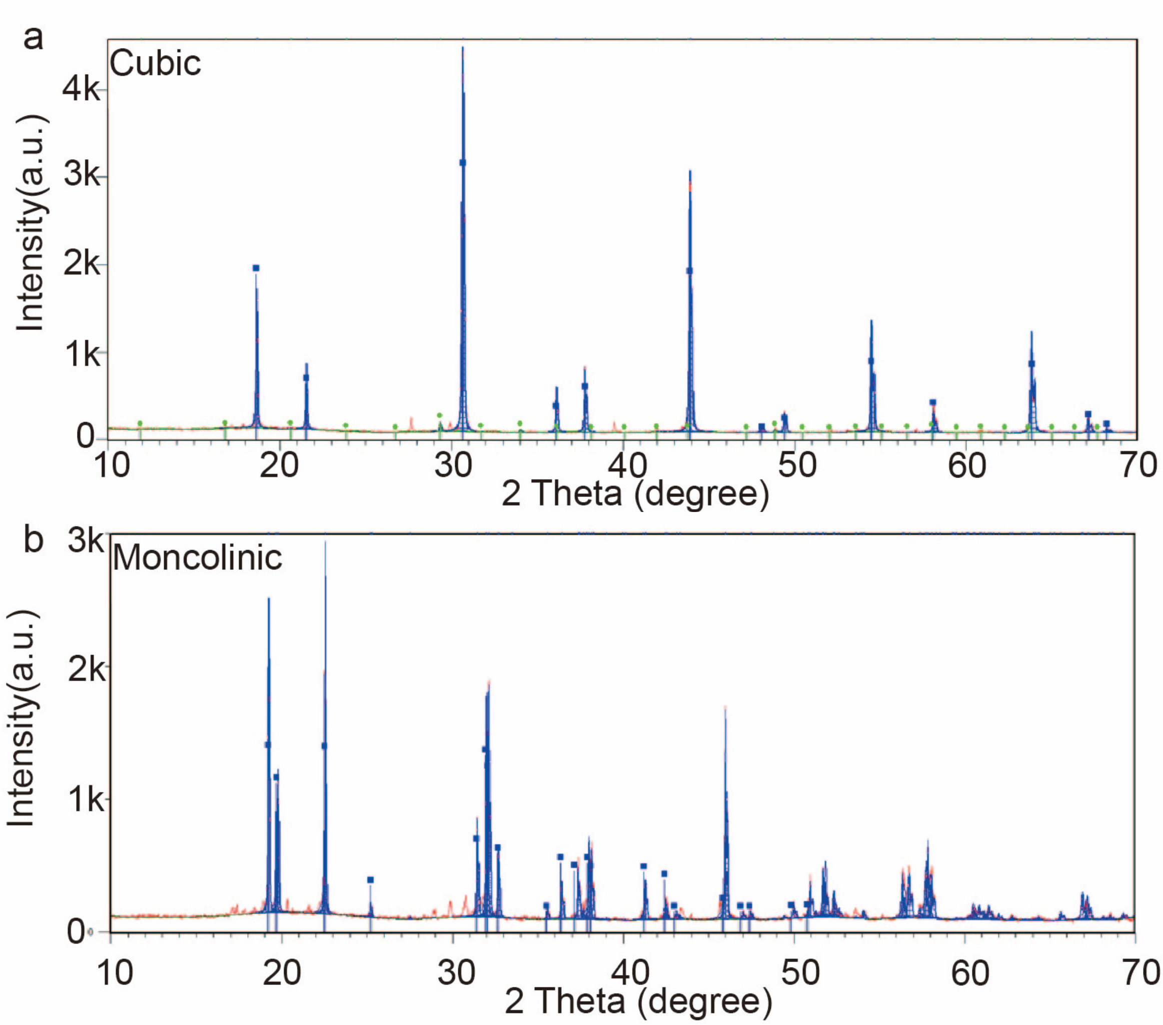

Figure S8. Rietveld-refined XRD patterns of $Na_{2.50}K_{0.50}GaF_6$:xEr^{3+} cubic phase and monoclinic phase.

Table S8. The occupation information of $Na_{2.50}K_{0.50}GaF_6$:xEr^{3+} cubic phase.

Atom	Wyck.	s.o.f.	x	y	z
F1	24e	0.969520	0.237316	0.000000	0.000000
K1	8c	1.000000	0.250000	0.250000	0.250000
NA1	4b	1.000000	0.500000	0.500000	0.500000

GA1	4a	0.908925	0.000000	0.000000	0.000000
Er1	4a	0.032999	0.000000	0.000000	0.000000

Table S9. The occupation information of $Na_{2.50}K_{0.50}GaF_6$:xEr^{3+} monoclinic phase.

Atom	Wyck.	s.o.f.	x	y	z
Ga1	2a	0.971776	0.000000	0.000000	0.000000
NA1	2b	0.834516	0.000000	0.000000	0.500000
NA2	4e	0.937824	0.500000	0.947672	0.240000
F1	4e	1.000000	0.088236	0.047957	0.223096
F2	4e	1.000000	0.704751	0.170017	0.038617
F3	4e	1.000000	0.154677	0.274840	0.935928
K1	2b	0.165484	0.000000	0.000000	0.500000
K2	4e	0.062176	0.500000	0.947672	0.240000
Er1	2a	0.028224	0.000000	0.000000	0.000000

Table S10. Rietveld refinement reliability parameters of cubic and monoclinic phases.

parameters	Cubic phase	Monoclinic phase
R expected	6.42215	6.69631
R profle	6.31763	7.531111
Weighted R profle	9.01751	0.70639
D-statistics	0.62513	0.49947
Weighted D-statistics	0.55031	0.46268
Goodness of Fit	1.40413	1.59885
